# Cumulative adult production and aerobic culture-dependent recovery of gut-associated bacteria in *Galleria mellonella* maintained on polymer-containing diets

**DOI:** 10.3389/finsc.2026.1960793

**Published:** 2026-10-07

**Authors:** Stephany Eileen Keferstein-Borges, Gabriela Alcalá-Gómez, Claudia Ivette Cisneros-Reyes, Jaime Alcalá-Gómez

**Affiliations:** 1 Laboratorio de Zoología, Departamento de Biotecnológicas y Ambientales, Universidad Autónoma de Guadalajara, Zapopan, Mexico; 2 Departamento de Biotecnológicas y Ambientales, Universidad Autónoma de Guadalajara, Zapopan, Mexico

**Keywords:** *Bacillus*, cultivable gut bacteria, greater wax moth, polyethylene, polyethylene terephthalate, polystyrene

## Abstract

Growing interest in insects capable of developing in the presence of synthetic polymers has created new opportunities to investigate host responses alongside cultivable gut microorganisms. This study evaluated cumulative adult production in *Galleria mellonella* maintained on a control diet or diets containing polyethylene terephthalate (PET), polystyrene (PS), or polyethylene (PE), together with the recovery of bacteria from the larval gut. Four dietary treatments control, PET, PS, and PE were established, each with five replicates of 15 initial larvae. Each experimental colony remained on its assigned diet until the initial cohort completed its life cycle and produced a second generation. Larvae from this offspring generation were selected for bacterial isolation, while the remaining individuals continued development for measurement of cumulative adult production. The isolates were characterized by sequencing the V4 region of the 16S rRNA gene and comparison using BLASTn, followed by genus-level taxonomic assignment. Cumulative adult production in the second generation differed among treatments (P = 0.0194). The control produced the highest mean number of adults (122.0 ± 56.3), whereas PET produced the lowest (41.8 ± 19.1) and was the only treatment significantly lower than the control. Fourteen bacterial isolates, selected as representatives of visually distinct colony morphotypes, were characterized; these isolates were not assumed to represent 14 distinct taxa or strains. Thirteen were assigned to *Bacillus* and one to *Psychrobacillus*. Overall, the dietary formulations evaluated were associated with variation in cumulative adult production and yielded an initial collection of cultivable gut-associated bacteria for subsequent bioprospecting studies.

## 1 Introduction

Plastic waste accumulation has become a major global environmental concern because of the sustained increase in synthetic polymer production, the persistence of these materials in the environment, and the limited efficiency of many conventional waste-management systems (1, 2). Alongside physical, chemical, and mechanical management strategies, plastic biodegradation has attracted increasing attention as a complementary approach, particularly because some microorganisms and enzymes can transform different polymers under specific conditions (3). In this context, several insect species and their associated microbiota have attracted interest as biological models for investigating interactions among polymeric substrates, host physiology, and gut microorganisms with potential biotechnological applications (4, 5).

*Galleria mellonella* (Lepidoptera: Pyralidae), commonly known as the greater wax moth, is naturally associated with honey bee colonies and combs. Beyond its biological relevance, it has been widely used as a model organism in studies of physiology, immunity, toxicity, and host–microorganism interactions (6, 7). Its value as an experimental model is further supported by its low cost, ease of handling, and suitability for assessing biological responses under controlled conditions (8). Interest in *G. mellonella* as a model for studying interactions with synthetic polymers increased following reports of larval ingestion and fragmentation of polyethylene and other polymers, as well as possible chemical transformation or biodegradation (9, 10). However, interpretation of these phenomena requires careful distinction among consumption, fragmentation, chemical transformation, and experimentally demonstrated degradation (11).

In *G. mellonella*, polyethylene (PE) and polystyrene (PS) are the most extensively studied polymers. Larval ingestion and fragmentation of these materials have been documented, together with changes in the polymers, effects on insect performance, and shifts in gut bacterial communities (4, 5, 12, 13). Sequencing-based studies have also described diet-associated changes in the bacterial communities of larvae maintained on HDPE-, LDPE-, PS-, or PVC-containing diets (14). Although viable bacteria have been recovered from larvae exposed to LDPE (15), microbial comparisons across polymer diets have largely relied on DNA-based community profiling. Culture-dependent isolation remains valuable because it provides viable microorganisms that can be preserved and evaluated directly in functional assays (16). Compared with PE and PS, polyethylene terephthalate (PET) has received considerably less attention in *G. mellonella*. A recent short-term study evaluated physical and chemical changes in PET following 48 h of exposure to the larvae (17), whereas activity toward PET has also been demonstrated using gut bacteria obtained from other insect species (18). Thus, information remains limited regarding population-level performance and cultivable bacteria associated with *G. mellonella* under dietary conditions involving PET, PS, and PE.

Against this background, this study aimed to describe cumulative adult production in *G. mellonella* maintained on a control diet or diets containing PET, PS, or PE and to recover cultivable gut-associated bacteria under aerobic culture conditions. The study provides a preliminary comparison of population-level performance across the four dietary formulations and a collection of viable isolates for subsequent functional evaluation. These two components were examined in parallel and do not constitute evidence of a causal relationship or polymer biodegradation.

## 2 Materials and methods

### 2.1 Insect source and experimental design

*G. mellonella* larvae were obtained as a single batch from PETMMAL, a commercial supplier of live feed for reptiles and amphibians located in Cuautitlán Izcalli, Estado de México, Mexico, and transported to the Zoology Laboratory at the Universidad Autónoma de Guadalajara, Zapopan, Jalisco, Mexico. Information on larval age, instar, source-colony generation, and feeding history before shipment was not available, and individual initial body mass was not recorded. During a 48-h acclimation period, the larvae were maintained on the same commercial waxworm feed subsequently used for the control treatment. Thereafter, 300 active larvae of visually similar size were selected, excluding conspicuously smaller or larger individuals.

Four dietary treatments were established: a commercial-feed control and three polymer-containing diets formulated with PET, PS, or PE. Each treatment comprised five independent experimental units of 15 larvae each, for a total of 300 larvae. For manual random allocation, the selected larvae were placed together in a plastic tray and allowed to move freely for 30 min to promote mixing. They were then transferred individually, without applying any further selection criteria, to the pre-labeled experimental units until each unit contained 15 larvae. Each experimental unit consisted of a glass Petri dish placed inside a 0.5-L plastic container covered with two layers of mesh secured with an elastic band to prevent larval escape.

All experimental units were maintained concurrently in the same laboratory space under a natural photoperiod. Periodic measurements with a digital thermohygrometer indicated a mean temperature of 24 ± 2 °C and mean relative humidity of 60 ± 8% (mean ± SD). These variables were not experimentally controlled or continuously recorded.

### 2.2 Diet preparation and feeding management

Feed portions were measured using a cylindrical 14.73-cm³ cup (3-cm internal height and 2.5-cm internal diameter). The commercial feed used was “WAX alimento para gusano de cera” (PETMMAL S.A. de C.V., Cuautitlán Izcalli, Estado de México, Mexico). Its label listed cereal flours, honey, glycerin, vitamins, and minerals but did not state their proportions or provide a guaranteed nutrient analysis. The proximate composition and energy content of the experimental diets were not determined.

PET from a clean bottle was grated into fine shavings, PS was crumbled into small beads, and PE protective material was cut with scissors into small fragments; particle sizes were not standardized. Each 14.73-cm³ ration contained either only commercial feed (control) or 2.95 cm³ of feed (20%) and 11.78 cm³ of PET, PS, or PE (80%). Because the materials differed in bulk density, the components were measured volumetrically and mixed before being provided to the larvae. They were not weighed; therefore, gram-based amounts and ratios are unavailable.

Experimental units were inspected daily, and the diets were provided *ad libitum*. A complete ration was added whenever the available material was visibly depleted. For the initial cohort, residual material was removed before replenishment until the onset of pupation. For the second generation, fresh rations were added without removing the existing material to avoid the accidental removal of eggs or small larvae. The number of replenishments and residual amounts were not recorded; therefore, cumulative feed intake and polymer exposure could not be quantified.

### 2.3 Second-generation larval sampling and adult monitoring

The initial cohort completed development and reproduction within each experimental unit. The offspring remained in the parental unit under the same assigned dietary treatment, without transfer or redistribution. All initial-cohort adults had died and been removed before any second-generation adults appeared and were excluded from the counts.

Second-generation larvae were first observed between 14 and 22 June 2023. On 17 August, 56–64 days later, one visually large larva was purposively selected from each replicate and weighed individually. Only larvae weighing 200–300 mg were included, yielding five larvae per treatment. This targeted criterion was used to standardize body size and obtain sufficient gut material for culture-based analysis. The remaining second-generation larvae continued development.

Second-generation adults remained in their units until natural death. During daily inspections, each dead adult was removed and counted once. Natural death served solely as the operational time point for enumeration rather than as a mortality or longevity endpoint. Monitoring lasted 105–134 days from the first observation of offspring and ended on 26 October, when no second-generation adults remained. Small third-generation larvae were observed, but none reached adulthood. Because individuals were not tracked separately, stage-specific durations, adult emergence dates, longevity, sex, mating, and oviposition were not recorded.

### 2.4 Larval dissection and sample preparation

Before dissection, larvae were surface-disinfected by immersion for 1 min in 0.1% active sodium hypochlorite (1,000 ppm), prepared by mixing 2 mL of commercial sodium hypochlorite (nominally 5%; Cloralex^®^) with 98 mL of sterile distilled water, followed by three rinses with sterile distilled water. The final rinse was not cultured as a control; therefore, complete removal of surface microorganisms was not verified.

Larvae were then euthanized and immobilized by immersion in water at 80 °C for 6 s, followed by exposure to −20 °C for 1 min. This procedure was adapted from entomological methods for larval killing and preservation using thermal shock and freezing (19, 20). Its potential effect on bacterial recovery was not assessed. Dissections were performed aseptically in a laminar-flow hood under a stereomicroscope using sterile instruments.

Most of the intestinal tract was excised from each selected larva, and the five intestinal samples from each treatment were pooled into a single composite sample in a 1.5-mL microcentrifuge tube. The pooled intestinal material was mechanically disrupted using a syringe needle to release the gut contents and suspended in 1,000 µL of sterile 0.9% NaCl solution (PiSA, Mexico) supplemented with 0.01 g of bacteriological peptone (1% w/v; MCD LAB, Mexico). Suspensions were vortexed for 10 s and incubated statically in closed 1.5-mL microcentrifuge tubes at 35 °C for 1 h, without atmospheric control, as a short pre-enrichment step. Because peptone provided a nutrient source, limited bacterial multiplication during this step could not be excluded; therefore, the microbiological findings were interpreted qualitatively. Two successive tenfold dilutions were then prepared from each suspension.

### 2.5 Culture media selection and bacterial cultivation

Five culture media were evaluated to recover cultivable bacteria associated with the gut contents of *G. mellonella*: lysine iron agar (LIA), triple sugar iron agar (TSI), bacteriological agar supplemented with yeast extract (YE), MacConkey agar (MAC), and brain heart infusion agar (BHI). The media panel was selected to include media with different nutrient and selective characteristics, considering the culture media available in the laboratory; BHI was additionally acquired for the study. The composition, pH, and intended role of each medium are provided in **Supplementary Table S1**. LIA and TSI were used only as plating media; their conventional biochemical reactions were not interpreted.

A preliminary trial showed that the undiluted suspensions produced confluent growth that prevented individual colonies from being distinguished; therefore, aliquots (100 µL) of the second serial dilution (10^-2^) were spread onto each medium using a Drigalski spatula. The plates were incubated at 35 °C under aerobic atmospheric conditions for 96 h and examined daily for the presence of visible colonies. Anaerobic, microaerophilic, and CO_2_-enriched conditions were not evaluated; consequently, bacterial recovery was restricted to the fraction cultivable under the aerobic conditions used. Colony numbers were not recorded.

Only plates containing macroscopically visible, spatially distinguishable colonies were used for bacterial isolation. Colonies exhibiting distinct morphologies were chosen based on differences in size, shape, margin, elevation, color, and surface appearance. Selected colonies were subcultured onto fresh medium to obtain pure cultures for subsequent morphological and molecular characterization.

### 2.6 Colony morphology and DNA extraction

Colony morphology was recorded before molecular characterization. Each bacterial isolate was inoculated into a test tube containing 10 mL of nutrient broth (**Supplementary Table S1**) and incubated at 35 °C for 7–10 days to obtain sufficient biomass for DNA extraction. Because isolate identities and growth characteristics were unknown at this stage, cultures were monitored visually and processed once visible sediment had accumulated; optical density was not measured. Genomic DNA was extracted using the phenol–chloroform method adapted from a previously described protocol (21), with modifications including proteinase K digestion, sequential extraction with phenol and chloroform:isoamyl alcohol (24:1), isopropanol precipitation, and 70% ethanol washing. DNA concentration and purity were measured using a NanoDrop spectrophotometer, and integrity was evaluated by electrophoresis on a 0.8% agarose gel stained with GelRed at 100 V for 20 min and visualized using a transilluminator.

### 2.7 16S rRNA amplification, sequencing, and taxonomic assignment

The V4 region of the 16S rRNA gene was amplified using primers 515F (5′-GTGCCAGCMGCCGCGGTAA-3′) and 806R (5′-GGACTACHVGGGTWTCTAAT-3′), as described by Caporaso et al. (22). The PCR was performed in a final volume of 30 μL containing 20 ng of DNA, 15 μL of 2× HSTM Mix, and 3 μL of each 10 μM primer (1 μM final concentration). Nuclease-free water and DNA from a laboratory bacterial sample were included as negative and positive controls, respectively. Amplification was performed in an MJ Research PTC-150 Thermal Cycler under the following conditions: initial denaturation at 94 °C for 5 min, followed by 35 cycles of denaturation at 94 °C for 45 s, annealing at 50 °C for 1 min, extension at 72 °C for 1 min, and a final extension at 72 °C for 10 min. PCR products with the expected size of 280–300 bp were analyzed by electrophoresis on a 1% agarose gel stained with GelRed. Five microliters of each PCR product were loaded onto the gel, and electrophoresis was performed at 90 V for 70 min.

Amplification products were purified with ExoSAP-IT™ and subsequently stored at −20 °C until sequencing. Bidirectional Sanger sequencing was carried out on a SeqStudio™ Genetic Analyzer System at the Laboratorio Nacional de Identificación y Caracterización Vegetal (LaniVeg), Centro Universitario de Ciencias Biológicas y Agropecuarias, Universidad de Guadalajara, Zapopan, Jalisco, Mexico. Forward and reverse chromatograms were visually inspected and assembled using CodonCode Aligner. Primer sequences and low-quality terminal regions were removed after assembly; therefore, the reported consensus sequences were shorter than the expected 280–300 bp complete amplicons. The resulting sequences were compared with the GenBank nucleotide database using BLASTn (23). Genus-level assignments considered percent identity, query coverage, E-value, and concordance among the highest-scoring matches. Identification was limited to the genus level when multiple species within the same genus showed comparable scores.

### 2.8 Statistical analysis

Cumulative adult production was defined as the total number of second-generation adults counted once in each experimental unit by the end of monitoring. Each replicate served as an independent unit of analysis, and the data were summarized by treatment as cumulative totals, means, and standard deviations.

The effect of diet on cumulative adult production was evaluated using one-way analysis of variance (ANOVA), with each replicate treated as an independent experimental unit. Model residuals were assessed for normality and homogeneity of variance using the Shapiro–Wilk and Levene tests, respectively. When ANOVA indicated a significant treatment effect, treatment means were compared using Tukey’s HSD *post hoc* test. All statistical analyses were conducted in R version 4.6.1 (24), with statistical significance set at *P* < 0.05.

Microbiological findings were summarized descriptively by composite sample of origin, culture medium of recovery, colony morphology used for isolate selection, and genus-level taxonomic assignment based on partial 16S rRNA gene sequencing and BLASTn comparison. Because the five guts were pooled before bacterial isolation, each treatment was represented by a single composite microbiological sample (n = 1). Individual isolates were not considered independent replicates, and isolate numbers were not used to infer bacterial abundance, diversity, or differences in bacterial composition among treatments.

## 3 Results

### 3.1 Culture media screening

Following inoculation of each medium with 100 µL of the 10^-2^ dilution, bacterial growth was recorded qualitatively as the presence or absence of visible colonies because colony counts were not performed (**Table 1**). No detectable growth was observed on LIA or MAC in any composite sample during the 96-h incubation period under aerobic atmospheric conditions at 35 °C. TSI showed visible growth from 48 h in the control and PS samples, whereas YE and BHI showed visible growth within the first 24 h in all four treatments. Based on these findings, TSI, YE, and BHI were selected for bacterial isolation. These observations represent only the fraction cultivable under the aerobic conditions used; anaerobic or microaerophilic growth and associated colony morphology were not evaluated.

**Table 1:** Qualitative bacterial growth from the second serial dilution (10^-2^) of composite gut samples during 96 h of aerobic incubation at 35 °C.

Culture medium	Control	PET	PS	PE	First detectable growth
Lysine iron agar (LIA)	–	–	–	–	Not detected
Triple sugar iron agar (TSI)	+	–	+	–	48 h
Yeast extract-supplemented bacteriological agar (YE)	+	+	+	+	24 h
MacConkey agar (MAC)	–	–	–	–	Not detected
Brain heart infusion agar (BHI)	+	+	+	+	24 h

Each dietary treatment was represented by one composite gut sample (n = 1). Only 100 µL aliquots of the second tenfold serial dilution (10^-2^) were evaluated in the definitive culture-media screening reported in this table. The presence (+) or absence (−) of visible colonies was recorded qualitatively; colony counts were not performed. “First detectable growth” indicates the earliest time at which visible colonies were observed for each culture medium among treatment samples showing positive growth.

### 3.2 Colony recovery and isolate selection

Fourteen isolates representing visually distinct colony morphotypes were selected for further characterization (**Table 2**). Six originated from the control composite sample, four from PS, and two each from PE and PET. Isolates from the control and PS samples were recovered from YE, BHI, and TSI, whereas those from the PE and PET samples were recovered only from YE and BHI. Because each treatment was represented by one composite gut sample, these values describe the distribution of selected colony morphotypes under the culture conditions used and do not represent estimates of bacterial abundance or diversity; therefore, no statistical comparisons were made among treatments.

**Table 2:** Distribution of bacterial isolates selected as representatives of visually distinct colony morphotypes by dietary treatment and culture medium.

Treatment	YE	BHI	TSI	Total selected isolates
Control	2	3	1	6
PS	1	2	1	4
PE	1	1	0	2
PET	1	1	0	2
Total	5	7	2	14

Values indicate the number of isolates selected as representatives of visually distinct colony morphotypes from one pooled sample comprising five larval guts per dietary treatment (n = 1 microbiological sample per treatment). These values do not represent total colony counts or estimates of bacterial abundance or diversity and were not statistically compared among treatments. YE, yeast extract-supplemented bacteriological agar; BHI, brain heart infusion agar; TSI, triple sugar iron agar; PS, polystyrene; PE, polyethylene; PET, polyethylene terephthalate.

### 3.3 Sequencing of the 16S rRNA gene V4 region and genus-level taxonomic assignment

Amplification of the 16S rRNA gene V4 region yielded products of approximately 300 bp for all 14 selected isolates. After assembly and removal of primer sequences and low-quality terminal regions from the bidirectional reads, final consensus sequences ranging from 244 to 253 bp were obtained. BLASTn searches against the NCBI nucleotide database supported genus-level taxonomic assignment. Thirteen isolates were assigned to *Bacillus*, whereas PET14 was assigned to *Psychrobacillus*. *Bacillus* isolates were recovered from all four dietary treatments: six from the control, four from PS, two from PE, and one from PET (**Table 3**).

**Table 3:** Genus-level taxonomic assignment of cultivable bacteria recovered from the gut of *G. mellonella* larvae based on sequencing of the 16S rRNA gene V4 region.

Treatment	Isolate	Sequence ID	Recovery medium	16S fragment length (bp)	Genus-level assignment	GenBank accession
Control	T1	GM_GUT_T1	YE	253	*Bacillus*	PZ779718
Control	T2	GM_GUT_T2	YE	253	*Bacillus*	PZ779719
Control	T3	GM_GUT_T3	TSI	253	*Bacillus*	PZ779720
Control	T4	GM_GUT_T4	BHI	253	*Bacillus*	PZ779721
Control	T5	GM_GUT_T5	BHI	253	*Bacillus*	PZ779722
Control	T10	GM_GUT_T10	BHI	253	*Bacillus*	PZ779723
PS	PS6	GM_GUT_PS6	BHI	253	*Bacillus*	PZ779724
PS	PS7	GM_GUT_PS7	BHI	244	*Bacillus*	PZ779725
PS	PS8	GM_GUT_PS8	YE	253	*Bacillus*	PZ779726
PS	PS9	GM_GUT_PS9	TSI	253	*Bacillus*	PZ779727
PE	PE11	GM_GUT_PE11	BHI	253	*Bacillus*	PZ779728
PE	PE12	GM_GUT_PE12	YE	253	*Bacillus*	PZ779729
PET	PET13	GM_GUT_PET13	BHI	253	*Bacillus*	PZ779730
PET	PET14	GM_GUT_PET14	YE	253	*Psychrobacillus*	PZ779731

Isolates listed for each dietary treatment were recovered from one pooled sample comprising five larval guts (n = 1 microbiological sample per treatment). Consequently, the taxonomic assignments are descriptive and should not be interpreted as treatment-level prevalence or differences in bacterial composition. YE, yeast extract-supplemented bacteriological agar; TSI, triple sugar iron agar; BHI, brain heart infusion agar; PS, polystyrene; PE, polyethylene; PET, polyethylene terephthalate; bp, base pairs. Taxonomic assignments were based on BLASTn comparison of partial 16S rRNA gene sequences against the NCBI nucleotide database.

### 3.4 Cumulative adult production under polymer-containing diets

Cumulative adult production in the second generation was summarized across the five independent experimental units within each dietary treatment (**Figure 1**). The mean ± SD per experimental unit was 122.0 ± 56.3 in the control, 60.4 ± 26.2 in PS, 64.0 ± 35.4 in PE, and 41.8 ± 19.1 in PET. The corresponding cumulative totals were 610, 302, 320, and 209, respectively.

**Figure 1:**
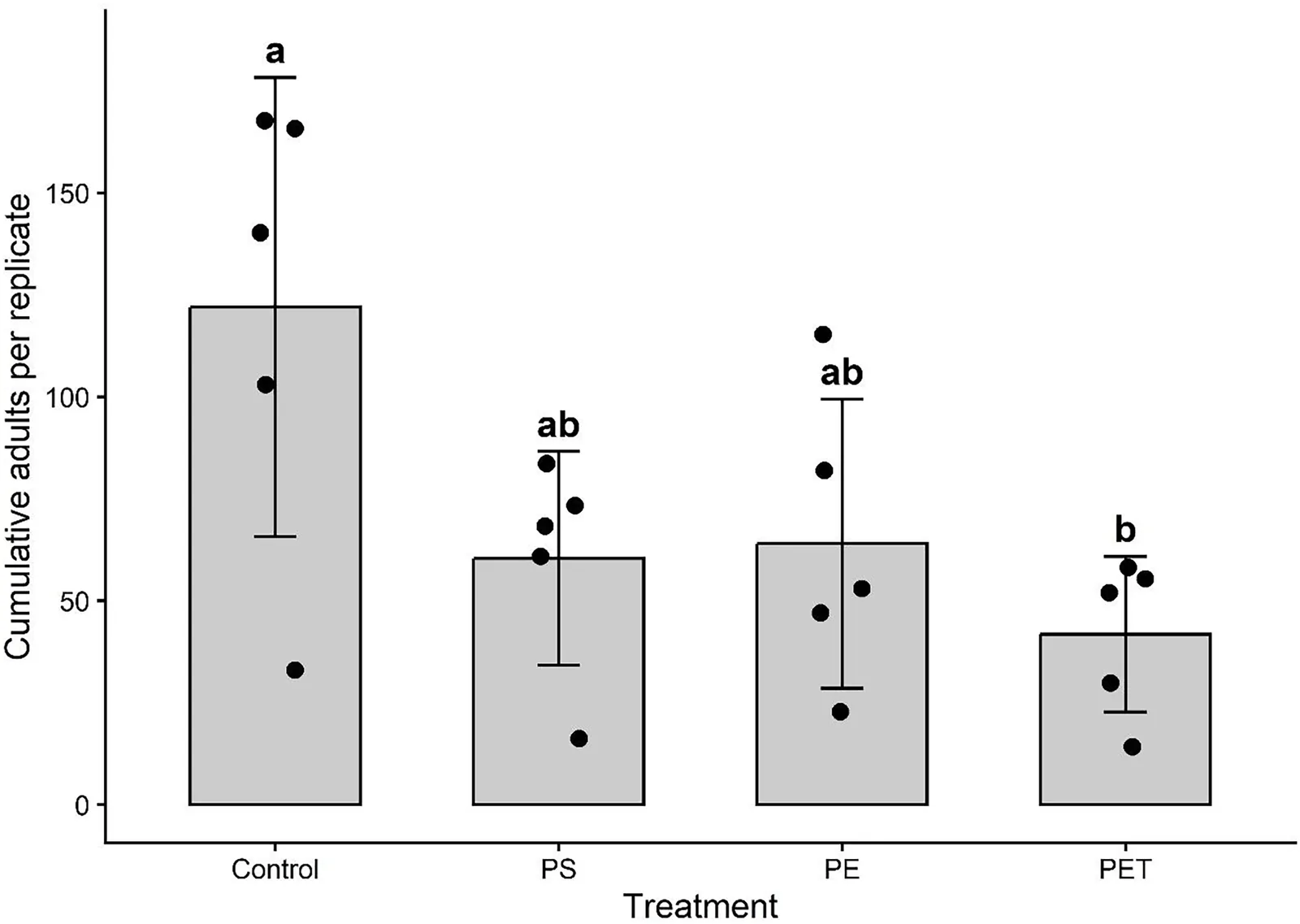
Cumulative adult production of second-generation *G. mellonella* under the control and polymer-containing diets. Bars represent treatment means, error bars indicate standard deviations (SD), and points represent individual experimental units (n = 5 per treatment). Different letters indicate significant differences among treatment means according to Tukey’s HSD *post hoc* test (*P* < 0.05). PS, polystyrene; PE, polyethylene; PET, polyethylene terephthalate.

One-way ANOVA indicated an overall difference among dietary treatments in cumulative adult production (*F*(3, 16) = 4.402, *P* = 0.0194). Tukey’s HSD *post hoc* test showed that the mean for PET was significantly lower than that of the control (*P* = 0.0163), whereas PS and PE did not differ significantly from the control (*P* = 0.0767 and *P* = 0.1016, respectively). Descriptively, treatment means were 50.5% lower for PS, 47.5% lower for PE, and 65.7% lower for PET relative to the control. Because commercial feed constituted 20% of the polymer-containing diets but 100% of the control diet, these results describe responses to the complete dietary formulations and do not allow the contribution of the lower commercial-feed proportion to be distinguished from that of polymer exposure.

## 4 Discussion

The results revealed a clear pattern in the population-level performance of *G. mellonella*: cumulative adult production was highest in the control, PET showed the lowest mean and the only statistically significant difference from the control, and PS and PE showed numerical reductions. In parallel, 14 cultivable bacterial isolates, predominantly assigned to *Bacillus*, were obtained across the four dietary conditions. Thus, this study provides preliminary information on insect responses to the dietary formulations evaluated and establishes a collection of viable microorganisms for further investigation. Because the two components were examined using different sampling frameworks, they should be interpreted as parallel findings rather than as evidence of a direct relationship between adult production and the bacteria recovered.

Cumulative adult production provides an integrated measure of colony performance because it reflects both reproduction by the initial cohort and development and emergence in the second generation. This approach allowed the final population-level outcome of each experimental unit to be evaluated, although it did not identify the individual contributions of oviposition, larval development, pupation, or adult emergence. The lower mean recorded for PET indicates that this formulation was less favorable than the control for sustaining second-generation adult production under the conditions evaluated. The numerical reductions observed for PS and PE suggest a similar pattern, but these treatments did not differ significantly from the control. Previous studies have reported that exposure of *G. mellonella* to certain plastics may be accompanied by changes in development, pupation, or egg production, emphasizing the importance of assessing life-cycle performance beyond immediate larval survival (25).

Diet composition is central to the interpretation of this pattern. In the polymer-containing treatments, PET, PS, or PE replaced 80% of the commercial feed by volume, whereas the control received commercial feed alone. Polymer exposure was therefore accompanied by a substantial reduction in the proportion of conventional nutrients. Because cumulative feed intake was not quantified, the respective contributions of nutrient availability and polymer exposure cannot be separated. Consequently, the observed differences should be attributed to the complete dietary formulations rather than specifically to the toxicity or metabolism of PET, PS, or PE. This interpretation is consistent with studies cautioning that ingestion or visible fragmentation of plastic materials does not demonstrate their nutritional utilization or bioassimilation by larvae (11, 26).

Bacterial recovery also showed a culture-medium-dependent pattern. YE and BHI yielded isolates from all four treatments, whereas TSI yielded isolates only from the control and PS samples; no detectable growth was observed on LIA or MacConkey agar. These differences demonstrate that the recovered fraction depended on medium composition and incubation conditions. Although a culture-dependent approach does not characterize the entire gut microbiota, it provides viable microorganisms that can be preserved and individually evaluated in functional assays (16). Because the five guts were pooled into one composite sample per treatment, isolate numbers represent selected colony morphotypes and not differences in bacterial abundance, diversity, or composition among treatments.

The predominance of *Bacillus* is consistent with the frequent occurrence of members of Bacillota in studies of insects exposed to plastic materials. Sequencing-based investigations of *G. mellonella* maintained on PE- or PS-containing diets have reported shifts in bacterial communities and the presence of *Bacillus*, *Serratia*, and other genera (4, 5, 13). More recently, 16S rRNA gene V1–V9 sequencing of gut bacterial communities from larvae maintained on diets containing HDPE, LDPE, PS, or PVC revealed diet-associated changes in community composition and predicted functions (14). These studies provide a community-level perspective, whereas the present work recovered viable isolates using an aerobic culture-dependent approach. The recovery of *Bacillus* from both the control and all three polymer-containing treatments indicates that its presence was not exclusive to any dietary formulation and cannot, by itself, be interpreted as evidence of polymer-related activity.

The isolate assigned to *Psychrobacillus* was recovered only from the PET sample. Although this finding expands the taxonomic range of the cultivable collection, the recovery of a single isolate and the absence of independent microbiological replicates preclude establishing a specific association with PET. Its relevance therefore lies primarily in its availability as a viable culture for subsequent functional comparison with the *Bacillus* isolates.

Partial sequencing of the V4 region of the 16S rRNA gene supported genus-level assignment of the isolates. After primer sequences were removed during sequence processing, the final consensus sequences ranged from 244 to 253 bp, explaining the difference from the approximate PCR product size before trimming. The resolution obtained did not support reliable species-level identification, particularly within *Bacillus*, which includes closely related species (27). The current assignments should therefore be considered an initial taxonomic characterization that may be refined using additional markers or genome-based approaches.

Mechanisms proposed in the literature provide a basis for the functional evaluation of this isolate collection. Previous studies have suggested the involvement of gut microorganisms and insect-derived enzymes in the modification of PE or PS (4, 5, 28, 29), whereas biological degradation of PET has been investigated primarily using specialized microorganisms and enzymes, including PETases and PET hydrolases (30, 31). Evaluations of insect-associated bacteria for activity toward PET have also recently begun (18). However, recovering cultivable bacteria does not demonstrate their ability to utilize or modify these materials. Establishing such activity will require assays with individual isolates and physicochemical analyses documenting verifiable changes in the polymers.

Overall, the findings show that the dietary formulations evaluated were associated with differences in cumulative adult production and that the larvae served as a source of bacteria cultivable under the conditions used. Future studies incorporating nutritionally comparable controls, quantitative feed-intake measurements, and stage-specific monitoring will allow the population-level response to be interpreted more precisely. In parallel, independent microbiological replicates, additional cultivation conditions, higher-resolution taxonomic identification, and functional assays will be required to determine the potential biotechnological relevance of the isolates. The present study therefore provides an experimental basis for further investigation without assuming a relationship between the two sets of findings or demonstrating biodegradation of PET, PS, or PE.

## 5 Conclusions

This study showed that the dietary formulations evaluated were associated with differences in cumulative adult production in *G. mellonella*, and cultivable gut-associated bacteria were recovered across all four dietary conditions. The PET treatment yielded the lowest cumulative adult production and was the only treatment that differed significantly from the control, suggesting that, under the conditions tested, this dietary formulation was less suitable for sustaining colony performance. Although PS and PE also produced numerical reductions, these differences were not statistically significant. These findings describe responses to the complete dietary formulations and do not distinguish the contribution of the lower commercial-feed proportion from that of polymer exposure.

The cultivable collection comprised 14 bacterial isolates, 13 of which were assigned to *Bacillus* and one to *Psychrobacillus*. The viable isolates constitute an exploratory culture collection that can be prioritized for future functional evaluation involving PET, PS, and PE. Their recovery should not be interpreted as evidence of treatment-level differences in bacterial composition or polymer biodegradation.

## Data Availability

The original contributions presented in this study are included in the article and **Supplementary Materials**. The partial 16S rRNA gene sequences generated in this study are available in NCBI GenBank (https://www.ncbi.nlm.nih.gov/genbank) under accession numbers PZ779718.1–PZ779731.1. Further inquiries can be directed to the corresponding author.
