# Supplementary material for: Cumulative adult production and aerobic culture-dependent recovery of gut-associated bacteria in Galleria mellonella maintained on polymer-containing diets
Source: Front Insect Sci. 2026 Oct 7;6:1960793. doi: 10.3389/finsc.2026.1960793 (PMC13683644; doi:10.3389/finsc.2026.1960793)
Supplement: Supplementary file 1 [file DataSheet1.pdf]

Supplementary Table S1. Composition, pH, and functional characteristics of the solid culture media and nutrient broth used in this study.

Culture medium	Manufacturer	Composition per liter	pH	Classification and role in the study
Lysine iron agar (LIA)	MCD LAB	Gelatin peptone, 5.0 g; yeast extract, 3.0 g; dextrose, 1.0 g; L-lysine, 10.0 g; sodium thiosulfate, 0.04 g; ferric ammonium citrate, 0.50 g; bromocresol purple, 0.02 g; bacteriological agar, 13.5 g	6.7 ± 0.2 at 25 °C	Differential medium included in the exploratory cultivation screen. Because it was used as a plating medium, the standard lysine decarboxylation, lysine deamination, and H_2S reactions were not interpreted.
Triple sugar iron agar (TSI)	MCD LAB	Casein peptone, 10.0 g; meat peptone, 10.0 g; meat extract, 3.0 g; yeast extract, 3.0 g; dextrose, 1.0 g; lactose, 10.0 g; sucrose, 10.0 g; ferric ammonium citrate, 0.3 g; sodium chloride, 5.0 g; sodium thiosulfate, 0.3 g; phenol red, 0.025 g; bacteriological agar, 12.0 g	7.4 ± 0.2 at 25 °C	Differential medium included in the exploratory cultivation screen. Because it was used as a plating medium, the standard carbohydrate fermentation, gas-production, and H_2S reactions were not interpreted.
Yeast extract agar (YE)	Laboratory-prepared using MCD LAB bacteriological agar and yeast extract	Bacteriological agar, 15.0 g; yeast extract, 5.0 g	7.0	Non-selective medium used to recover bacteria capable of growing with yeast extract as the nutrient source under the aerobic conditions evaluated.
MacConkey agar (MAC)	MCD LAB	Pancreatic digest of gelatin, 17.0 g; peptic digest of animal tissue, 1.5 g; pancreatic digest of casein, 1.5 g; lactose, 10.0 g; bile salts, 1.5 g; sodium chloride, 5.0 g; crystal violet, 0.001 g; neutral red, 0.03 g; bacteriological agar, 13.5 g	7.1 ± 0.2 at 25 °C	Selective and differential medium included to screen for the growth of Gram-negative enteric bacteria and distinguish lactose-fermenting colonies.

Brain heart infusion agar (BHI)	BD Bioxon™	Brain–heart infusion, 8.0 g; casein peptone, 16.0 g; meat peptone, 5.0 g; sodium chloride, 5.0 g; disodium phosphate, 2.5 g; dextrose, 2.0 g; agar, 13.5 g	7.3 ± 0.2 at 25 °C	Enriched, non-selective medium included to support a broad range of cultivable bacteria, including nutritionally demanding organisms capable of growing under the aerobic conditions evaluated.
Nutrient broth	BD Bioxon™	Gelatin peptone, 5.0 g; beef extract, 3.0 g	6.9 ± 0.2 at 25 °C	General-purpose liquid medium used to generate bacterial biomass before genomic DNA extraction.

Note: Manufacturer-reported formulations and pH values are presented for LIA, TSI, MAC, BHI, and nutrient broth. YE was prepared in the laboratory using 15 g of bacteriological agar and 5 g of yeast extract per liter, with a final pH of 7.0. No blood or other enrichment supplements were added to BHI. The five solid media were used as plating media and incubated at 35 °C under aerobic atmospheric conditions for 96 h. The conventional biochemical reactions of LIA and TSI were not interpreted. Nutrient broth was used only to generate bacterial biomass before genomic DNA extraction. Anaerobic, microaerophilic, and CO_2-enriched conditions were not evaluated for the plating media.
